# Gallic Acid-Containing Gelatin-Based Nonwoven Mat with Synergistic Photodegradation and Photoindication Function for Reducing Nicotine

**DOI:** 10.3390/polym13234245

**Published:** 2021-12-03

**Authors:** Meng-Yi Bai, Ting-Teng Wang

**Affiliations:** 1 Graduate Institute of Biomedical Engineering and Biomedical Engineering Program, Graduate Institute of Applied Science and Technology, National Taiwan University of Science and Technology, TR-917, AAEON Building, No.43, Keelung Rd., Sec.4, Da’an Dist., Taipei City 10607, Taiwan; stanley777100@gmail.com; 2 Adjunct Appointment to the National Defense Medical Center, Taipei City 11490, Taiwan

**Keywords:** cigarette, smoke, fluorescence, gelatin, nonwoven mat, UV

## Abstract

Cigarette smoking is a popular habit that has negative health consequences for populations. In this study, we developed a gallic acid-containing, gelatin-based nonwoven mat with photodegradation and photoindication functions. This could react with sidestream cigarette smoke and simultaneously inhibit the activity of the microbe growth in the air. The results of a fluorescence emission spectrum evidenced this photoindication function. Neither the nicotine nor gallic acid showed a redshift emission spectrum. However, the emission spectrum of the nonwoven mat exhibited the redshift and increased in intensity after absorbing the sidestream cigarette smoke. In this spectral evidence, the natural polymer played a key role in the photoindication function’s display because it could dissolve the nicotine of the sidestream cigarette smoke and cause it to react with the gelatin structure. The high performance liquid chromatography–mass spectroscopy results indicated that the gallic acid and ultraviolet (UV) light enhanced the absorption of nicotine and nicotine-like derivatives, which were dissolved by the Tween 80 of nonwoven mat. The liquid paraffin and Tween 80 could oxidize, dehydrogenate, and demethylate the nicotine that was absorbed by the gelatin nonwoven mat. In conclusion, the nonwoven mat developed in this study provided the functions to filter the nicotine of sidestream smoke and activate the photoindication property by absorbing 365-nm UV light.

## 1. Introduction

Cigarette smoking is a popular habit that causes negative health consequences worldwide, including pulmonary diseases, cardiovascular diseases, and cancer [1]. When one smokes a cigarette, nicotine in the mainstream smoke is breathed into the lungs and the sidestream smoke pollutes the nearby air [2]. Some medical research has suggested that nicotine is the major addictive component in tobacco and inhaling excessive nicotine can cause limb peripheral vasoconstriction, rapid heartbeat, elevated blood pressure, platelet aggregation, cardiovascular blockage, stroke, and other cardiovascular diseases [3]. Nicotine is the major compound emitted in cigarette smoke and is soluble in chloroform, ether, ethanol, oil, and water. The common nicotine-like derivatives include parent nicotine (163), nornicotine (149), nicotine-N-oxide (179), cotinine (177), and trans-3′-hydroxycotinine (193); these are the major components of nicotine [4].

Considering the deleterious influence of cigarette smoking on human health as well as the importance of lung health given the risk of COVID-19 infection, we developed a nonwoven mat that can simultaneously provide the functions of indicating air pollutant levels and purifying the air. Moreover, to apply this nonwoven mat in daily life, we designed a small, compact, wearable device for the user to clip onto cloth, rather than the type of bulky air purifier commonly used in household environments. The benefit of this new compact wearable device is that it can help immobilize the porous and thin nonwoven mat and provide a visible indication of specific air pollutant levels in situ. When the mat user perceives a color change in the nonwoven mat, they can either actively leave the sidestream smoke area or temporarily remain, using the device to help reduce the pollutant levels until the user can relocate to a place with cleaner air. A piece of light and thin nonwoven material is the basis for providing the photodegradation and photoindication functions; thus, this material should be biodegradable and environmentally friendly. Researchers have discovered that when some polyphenoic acids, such as ellagic acid [5,6], are directly bound to certain molecules, their molecular configuration changes and markedly enhances their ultraviolet (UV) absorption (maximum absorption wavelength 280 nm). Furthermore, ellagic acid then emits a fluorescence spectrum at approximately 400–450 nm. To strike a balance between biodegradability and ease of electrospinning protocol, we adopted a natural polymer gelatin, which is a tasteless, colorless, solid substance derived from collagen, as the main constituent of the electrospun nonwoven mat due to its array of advantages, such as biological origin, nonimmunogenicity, biodegradability, and biocompatibility [7,8]. The biodegradability of gelatin could be used to dispose of the nonwoven mats that adsorb nicotine. In some studies, researchers have reported that gelatin nanofibers exhibit outstanding properties of surface area and functionality, rendering them suitable in the development of biomimicking artificial extracellular matrices, wound dressing materials with antibacterial properties, and drug delivery matrices [9,10]. We followed Huang et al. in screening a series of polyphenol compounds to render the photoindication and photodegradation functionalities of the nonwoven mat. Among the polyphenolic compounds used, gallic acid reportedly exerts numerous therapeutic effects, including antimicrobial, antioxidant, and anticancer activities [11,12]. In some studies, gallic acid was used as an antifungal agent [13] and an antibacterial drug [14]. It is also widely noted in the plant kingdom and is largely found in free-form or as a derivative in various food sources, such as nuts, tea, grapes, and sumac [15,16]. Other sources include gallnuts, oak bark, honey, assorted berries, pomegranate, mango, and other fruits, vegetables, and beverages. Therefore, the proposed gallic acid-containing, gelatin-based nonwoven mat was designed and fabricated using the electrospinning technique. In this study, a series of photoluminescence spectra and high-performance liquid chromatography–mass spectroscopy (HPLC–MS) were used to develop and verify a nonwoven mat possessing photodegradation and photoindication functions. The porous membrane composed of nanofibers could react with sidestream smoke and inhibit the activity of microbes.

## 2. Experimental Section

### 2.1. Materials

Gelatin (100 g; solid; fish skin; G7041-100G), poly(ethylene oxide) (250 g; powder; average Mv ~900,000; 189456-250G), and gallic acid (100 g; powder; 97.50–102.50% (titration); G7384-100G) were purchased from Sigma-Aldrich (St. Louis, MO, USA); Tween 80 (500 mL; liquid; HLB (Hydrophile–Lipophile Balance Number) = 15; HB5I73) was obtained from Emperor Chemicals (Hangzhou, Zhejiang, China); and liquid paraffin (500 g; liquid; specific gravity 44 > 0.85; NO. 751103) was sourced from Santoku Chemicals (Chuo-ku, Tokyo, Japan).

### 2.2. Preparation of Gallic Acid-Containing, Gelatin-Based Nonwoven Mat

The gallic acid-containing, gelatin-based nonwoven mat was fabricated using a stock solution containing fish gelatin (fG), gallic acid (g), and nicotine-dissolving recipients, namely liquid paraffin (lp) and Tween 80 (T). The single-spinneret electrospinning technique, which involved a high voltage power supply (205B-20R; Bertan, You-shang Technical Corp, Kaohsiung City, Taiwan (R.O.C.)) and syringe pumps (NE-300, Just Infusion, Farmingdale, NY, USA), was used to generate the electrospun gallic acid-containing, gelatin-based nonwoven mat.

To determine the optimal recipe for fabrication of the gallic acid-containing, gelatin-based nonwoven mat, two types of nonwoven mats were prepared using different nicotine-dissolving recipients. One nicotine-dissolving recipient was liquid paraffin, which was dissolved with PEG (polyethylene glycol) (Sigma) by using formic acid (Sigma), and the other was dissolved using hot deionized (DI) water (1:2 measure). First, the fish gelatin was weighed and then dissolved using formic acid (120 mg dissolved in 200 μL formic acid) and subsequently stirred together with the liquid paraffin–PEG mixture (150 μL/20 mg) or Tween 80 solution (33 μL Tween 80, stock solution mixed with 67 μL DI water). Second, the 100 μL of 0.587 M gallic acid solution, which was dissolved with ethanol (100 mg dissolved in 1 mL ethanol), was pipetted to the previous mixture and stirred well. Finally, the aforementioned mixture was fed by a syringe pump and electrospun to generate two distinct types of gallic acid-containing, gelatin-based nonwoven mats.

### 2.3. Adsorption Measurement of Nicotine from the Sidestream Cigarette Smoke

The nonwoven mat was weighed and divided into three equal parts that were placed in a container full of sidestream smoke, as illustrated by the schematic in Scheme S1. The space of the container was segmented into two parts by using a cardboard barrier; one of the chambers was equipped with the cigarettes and 365-nm UV flashlight and the other one only contained the cigarettes. Then, gallic acid-containing, gelatin-based nonwoven mats were fastened to the roof of each chamber with a retaining clip. After the sidestream smoke was vented through interstices of the box, the test samples, which were named according to their ingredients and test parameters, were fixed and tested. The experimental groups were abbreviated as follows: fGglp, F–fGglp, UV–F–fGglp, fGgT, F–fGgT, and UV–F–fGgT (fG: fish gelatin, g: gallic acid, lp: liquid paraffin, T: Tween 80, F: fumigated, UV: 365-nm UV light).

### 2.4. Experimental Analyses

The fiber structure of the nonwoven mats was observed using scanning electron microscopy (SEM; JSM-6500F, JEOL), and all relevant statistical results were calculated using Image J (National Institutes of Health, Bethesda, MD, USA). Fluorescence emission spectra of the nonwoven mats were fumigated by cigarettes in Scheme S1 and acquired using a fluorescence spectrophotometer (Jasco, FP-8500). The nonwoven mats were set to a measurement block and excited by 365 nm UV light. Fluorescence emission spectra were obtained from a solid matrix because we had hoped to simulate the actual situation in the device and avoid the quenching property of the liquid sample. To determine the amount of nicotine adsorption and the degradation by-product, the experimental samples were loaded into individual bottles, each containing 4 mL of methanol, and then the nonwoven mats were soaked in methanol for 4 h to dissolve the nicotine and degradation by-products. After 4 h, the methanol extract solution in every bottle was filtered (0.22-μm filters) and submitted for HPLC–UV. In the HPLC–UV spectrum, the mixture was separated into individual components and then calculated based on the integral area of the UV absorption peak. Finally, the integral area was transformed to the absorption dose by using the standard curve and Equation (1).

(1) $$\mathrm{Absorption}\;\mathrm{dose}=\frac{C\left(\mathrm{dilution}\;\mathrm{ratio}\right)\left(\mathrm{amount}\;\mathrm{of}\;\mathrm{methanol}\right)}{1000}$$

Equation (1): The transformed equation of absorption dose, where C is the concentration that was calculated using the standard curve (ppm).

To identify the gallic acid, gelatin, and nicotine derivatives, the nonwoven mats were soaked in DI water, and the solutions were then filtered using 0.22-μm filters. The HPLC–MS results were provided by the National Tsing Hua University Precision Instrument Center (HPLC–MS, VARIAN 901-MS), which was commissioned to conduct tests on the solutions.

To secure the thin and lightweight nonwoven mat, we designed a type of wearable equipment that included a powerful yet compact blower (30 mm × 30 mm × 3.8 mm, 9500 rpm ± 20%, 88.18 Pa, 72 mA; Invni Tech Developing Corp.), as shown in Scheme S2, a UV light-emitting diode (5 mm, 365–370 nm, 3.6–3.8 V, 20 mA; Koodyz Technology), and a piece of gallic acid-containing, gelatin-based nonwoven mat. The wearable equipment could create an indraft to filter sidestream smoke though the nonwoven mat, and then the filtrated pollute could be captured, dissolved, and used to derive the fluorescence emission for photoindication. Two clean boxes were prepared with content agar plates, as shown in Table S4. One of the boxes was prepared with the wearable equipment and nonwoven mat, which could flow the air and filter the pollute, and the other was prepared with the same type of blower to flow the air and blow the dust to the surface of agar. Then, the plates were cultured for 48 h in the incubator.

## 3. Results and Discussion

**Figure 1:**
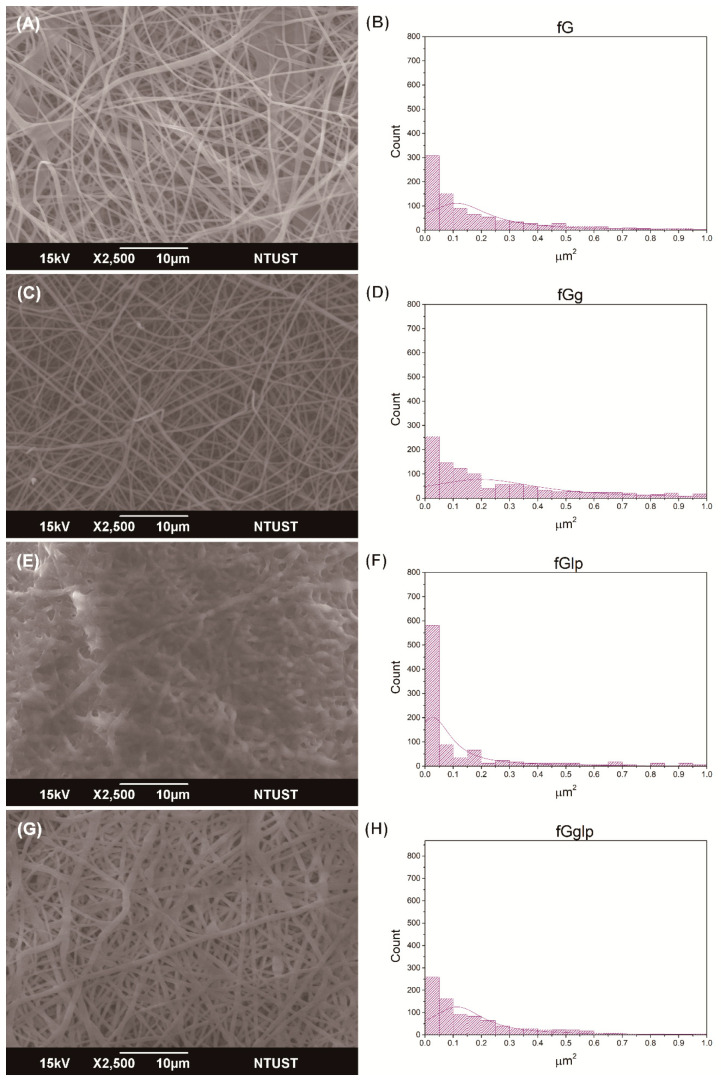
The SEM images of indicative nonwoven mat. (**A**,**B**) fG; (**C**,**D**) fGg; (**E**,**F**) fGlp; (**G**,**H**) fGglp. (fG: fish gelatin, g: gallic acid, lp: liquid paraffin).

**Figure 2:**
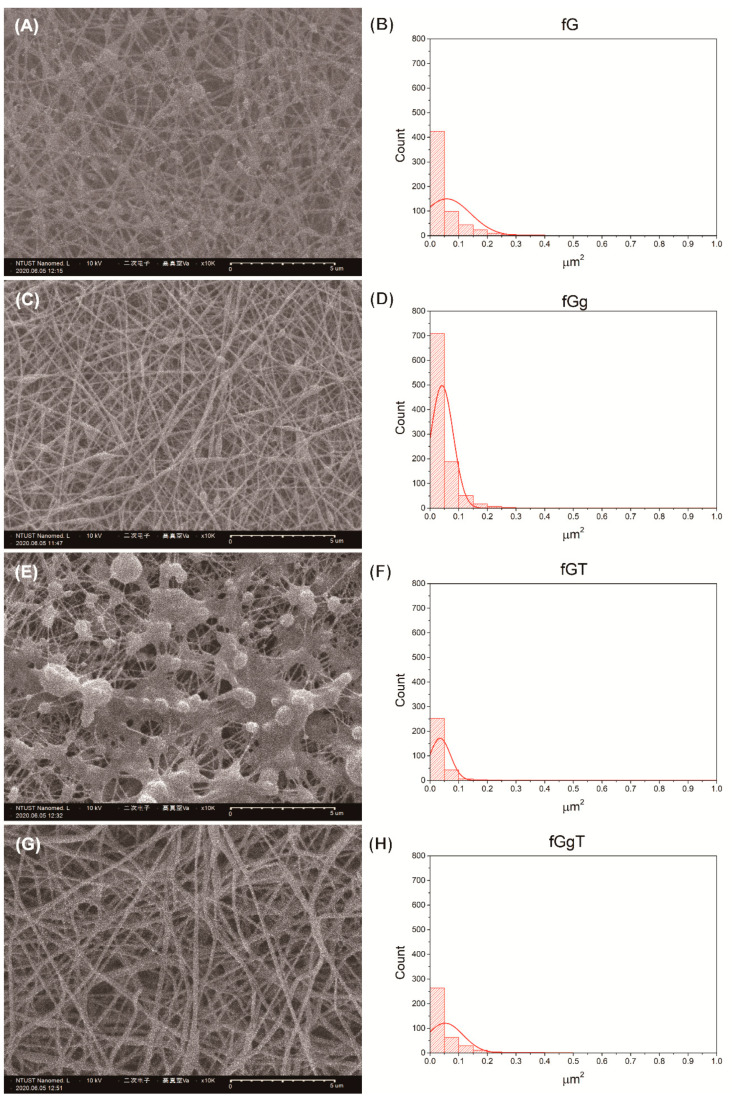
The SEM images of indicative nonwoven mat. (**A**,**B**) fG; (**C**,**D**) fGg; (**E**,**F**) fGT; (**G**,**H**) fGgT. (fG: fish gelatin, g: gallic acid, T: Tween 80).

The structure of the nonwoven mat was evaluated based on the SEM results. The porous structure could increase the contact area, allowing the mat to obstruct more sidestream smoke. Although the nicotine-dissolving polymer helped provide the photoindication property, it could clog the pores and decrease the fiber morphology formation, causing suboptimal porosity. By contrast, gallic acid was a noteworthy material that improved the gelatin fiber structure and enhanced the porosity regardless of whether paraffin or Tween 80 was used (Tables S1 and S2). This suggests that the addition of gallic acid not only induces the photoindication function but also alleviates the side effects of the nicotine-dissolving recipients (i.e., paraffin or Tween 80). In the SEM results, the major pore size distributions that were expected to filter and absorb the pollution of sidestream smoke were as small as 0.05 µm^2^. Figure 1E illustrates that liquid paraffin coated the fibers and clogged up the pores, destroying the porous structure and generating pore size distributions of approximately 0.025–0.05 µm^2^ (Figure 1F). The width of fibers and the porosity was markedly superior when the nonwoven mat was prepared using gelatin, gallic acid, and liquid paraffin recipe pairs, as shown in Figure 1G,H. Before the electrospinning, the liquid paraffin was mixed with the gelatin solution, which increased the viscosity of the mixture and simplified the generation of the electrospun nonwoven mat. In contrast, when Tween 80, rather than liquid paraffin, was used as the nicotine-dissolving polymer, the resultant nonwoven mat was composed of fibers but exhibited a bead morphology that covered the pores, markedly reducing the porosity (Figure 2E,F). Gallic acid could be used to provide photodegradation functionality and strike a balance between the viscosity of the solution and the integrity of the electrospun nonwoven mat. Therefore, the generation of beads was greatly reduced by adding gallic acid when the nonwoven mat was prepared using the stock solution (consisting of gelatin, gallic acid, and Tween 80). The results of the gallic acid-containing nonwoven mats could not only ensure the photoindication function but also enhance the fiber-forming property of the gelatin, as shown in Figure 2G,H.

**Figure 3:**
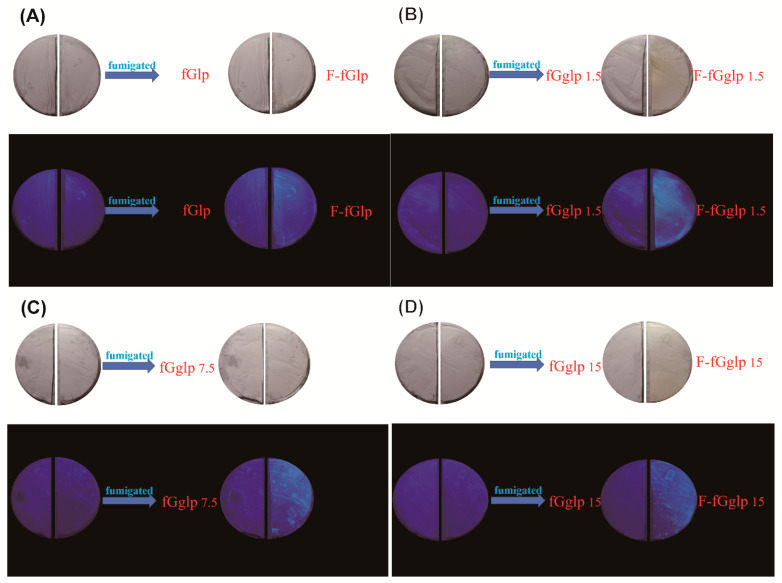
The fluorescence emission pictures of fGglp indicative nonwoven mat system with different concentrations of liquid paraffin: (**A**) 10 μL, (**B**) 15 μL, (**C**) 75 μL, and (**D**) 150 μL. (fG: fish gelatin, g: gallic acid, lp: liquid paraffin, F: fumigated).

**Figure 4:**
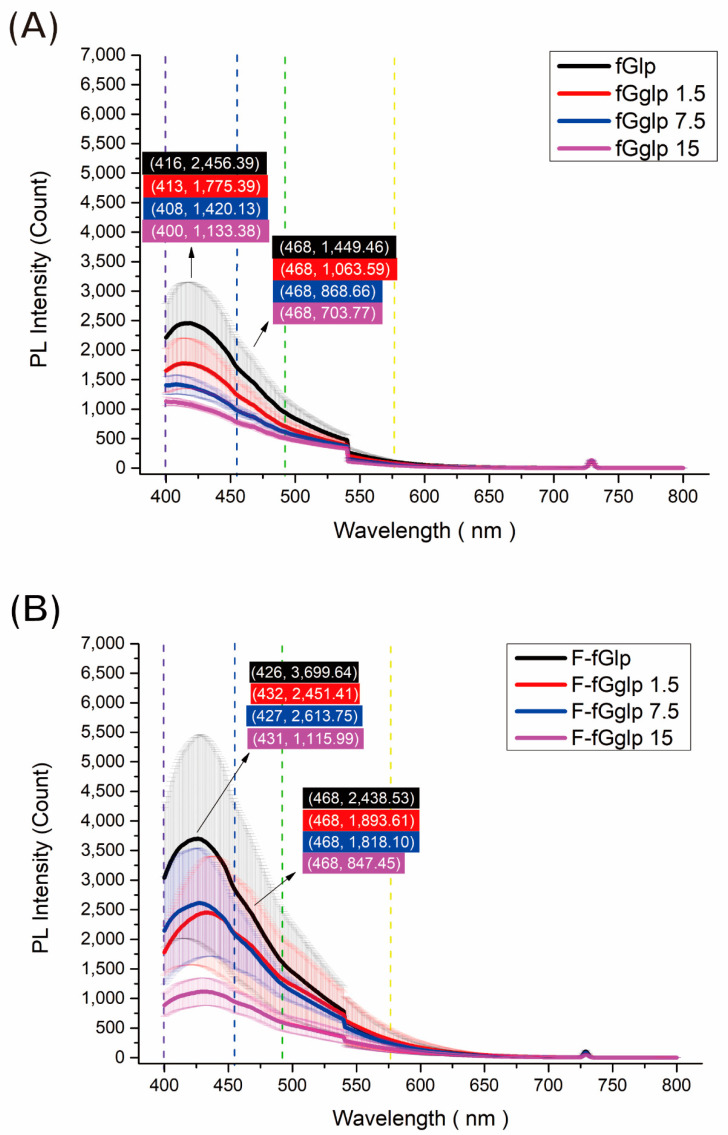
The fluorescence emission spectrum. (**A**) Non-fumigated, (**B**) fumigated. (fG: fish gelatin, g: gallic acid, lp: liquid paraffin, F: fumigated).

**Figure 5:**
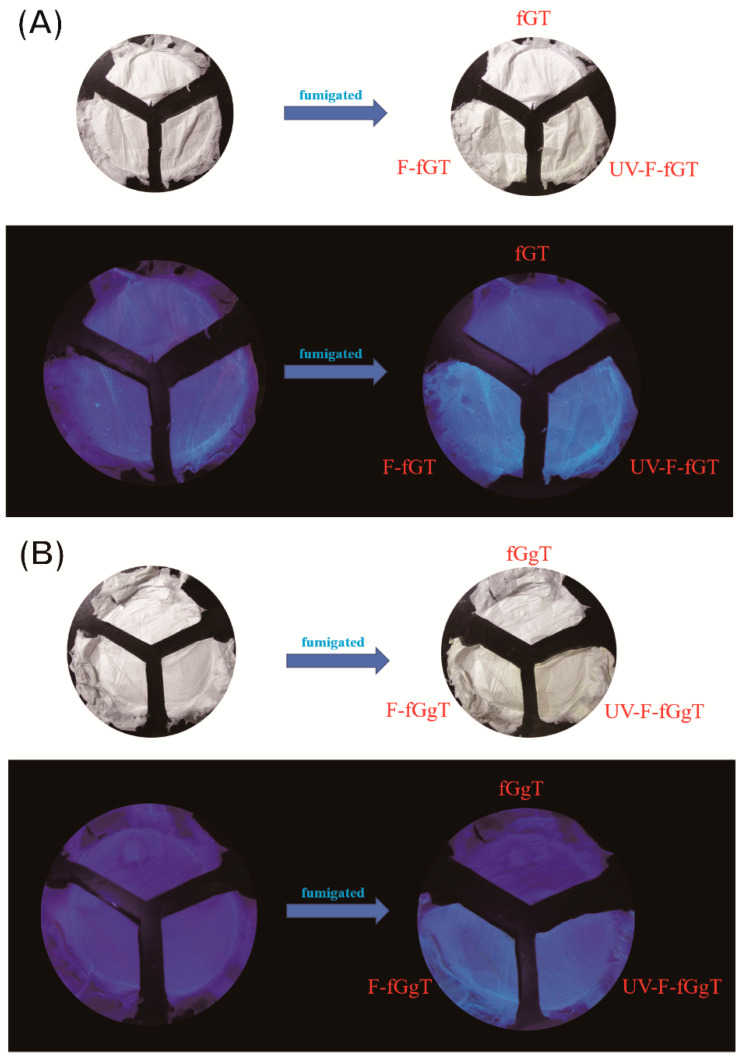
The fluorescence emission pictures of (**A**) indicative nonwoven mat and (**B**) gallic acid-containing indicative nonwoven mat. (fG: fish gelatin, g: gallic acid, T: Tween 80, F: fumigated, UV: 365 nm).

**Figure 6:**
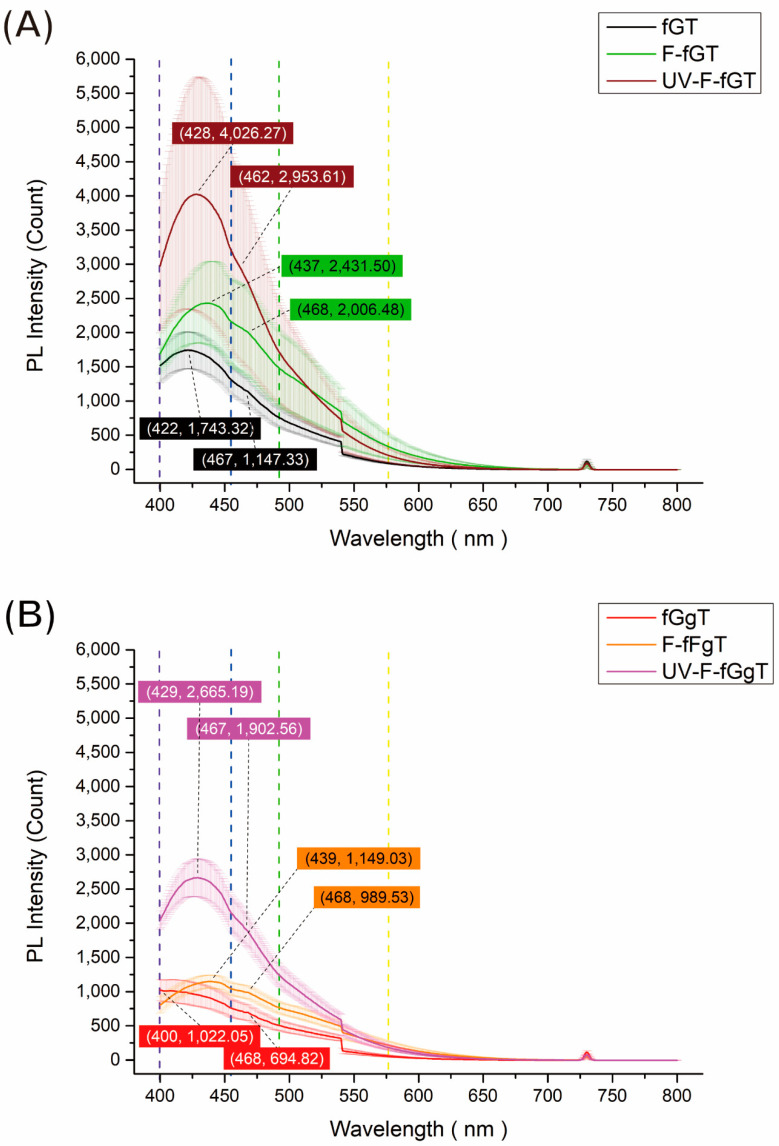
The fluorescence emission spectrum of (**A**) indicative nonwoven mat and (**B**) gallic acid-containing indicative nonwoven mat. (fG: fish gelatin, g: gallic acid, T: Tween 80, F: fumigated, UV: 365 nm).

The fluorescence emission spectra results could be used to evidence the process of photoindication. The nicotine-dissolving polymer materials (i.e., liquid paraffin and Tween 80) were the key components in the nonwoven mat, responsible for adsorbing the nicotine for fluorescence emission. To observe the change in fluorescence property between nicotine, nicotine-dissolving polymer materials, and 365 nm UV light, the equal parts of nonwoven mats were recorded to measure the disparity in 365 nm UV. The equal parts of nonwoven mats could emit fluorescence after the fumigation step. Then, the nonwoven mats were used to detect the distribution of emission wavelength and the disparity of intensity by fluorescence spectrophotometer, as shown in Figure 3, Figure 4, Figure 5 and Figure 6. In Figure 4, the quantitative emission spectra acquired from the fGglp (fish gelatin/gallic acid/liquid paraffin) nonwoven mat with different proportions of added paraffin proved the fluorescence intensity and wavelength before and after the sidestream smoke was absorbed. The cigarette smoke caused a purple emission light redshift at approximately 10–30 nm and simultaneously enhanced the intensity of the blue emission light at 468 nm. Figure 6 indicates the emission spectrum acquired from the fGgT (fish gelatin/gallic acid/Tween 80) nonwoven mat system, which was irradiated by a UV light (365 nm, UV absorption maximum wavelength of gallic acid) and simultaneously adsorbed the sidestream smoke to confirm the effect of UV irradiation on the activation of photoindication and the photodegradation property. The purple emission peak (400 nm) exhibited redshift at approximately 29 nm before and after UV exposure. Notably, the fluorescence did not disappear after the fumigation; conversely, the emission peak intensity at 429 and 467 nm was considerably enhanced (Figure 6B). This result reveals that the nicotine-dissolving polymer (i.e., Tween 80) played a key role in dissolving the sidestream-smoke nicotine, possibly performing a similar function to that of the ellagic acid, which altered the conformation of the chemical structure after reacting with some small molecules. When the nonwoven mat was irradiated by 365-nm UV light and adsorbed the sidestream smoke, the fluorescence emission was activated and markedly greater (see Figure 6B, pink curve) than that of the nonexposed nonwoven mat (see Figure 6B, orange curve).

**Figure 7:**
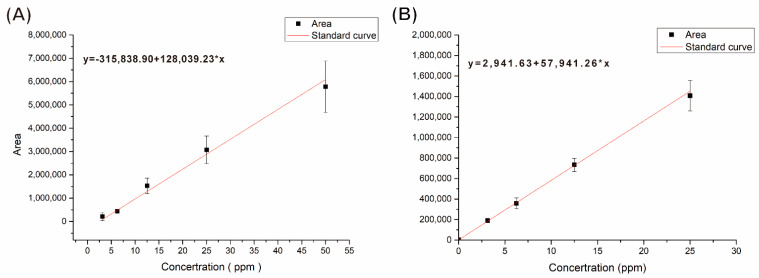
The standard curves that were plotted depending on integral area and concentration (ppm). (**A**) Gallic acid, (**B**) nicotine.

**Figure 8:**
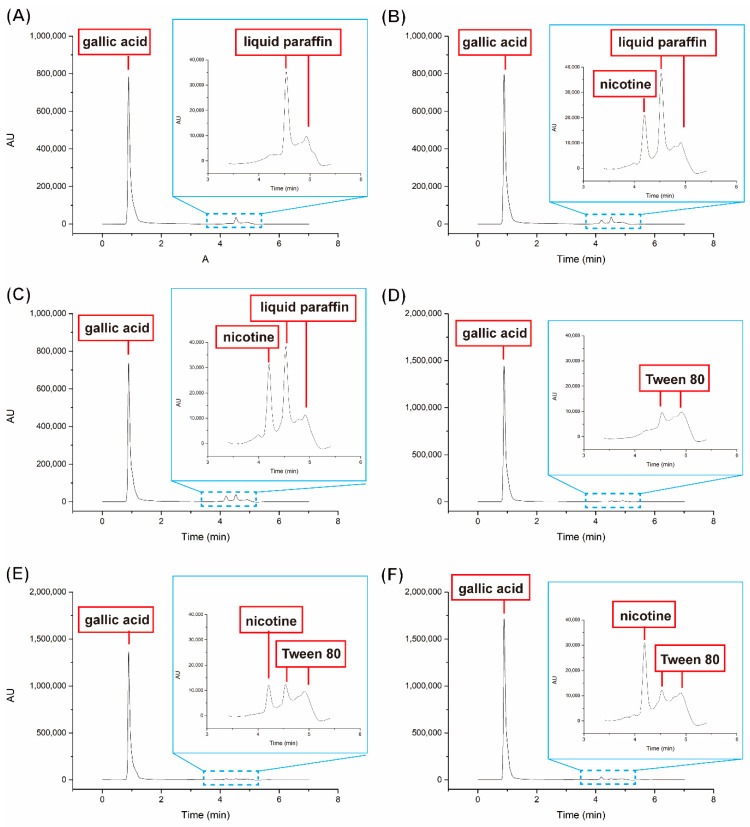
The HPLC–UV spectrum of (**A**) fGglp, (**B**) F–fGglp, (**C**) UV–F–fGglp, (**D**) fGgT, (**E**) F–fGgT, (**F**) UV–F–fGgT. (fG: fish gelatin, g: gallic acid, lp: liquid paraffin, T: Tween 80, F: fumigated, UV: 365 nm).

HPLC–UV was used to determine the efficiency of liquid paraffin and Tween 80 in the nonwoven mat for enhancing nicotine capture. The amount of nicotine and gallic acid was determined using the standard curves that were established using the integration area of the UV peak absorption versus concentration (ppm; Figure 7). As can be seen in the figure, two equations were derived from the standard curves for gallic acid and nicotine, respectively. As the components of our nonwoven mats included gallic acid, Tween 80, liquid paraffin, and the adsorbate (i.e., nicotine), we detected the releases of those components in the fGglp (fish gelatin/gallic acid/liquid paraffin) and fGgT (fish gelatin/gallic acid/Tween 80) nonwoven mats. In Figure 8, the analyte peak elutions at 0.89, 4.20, and 4.53 min in the HPLC–UV spectra were attributed to gallic acid, nicotine, and liquid paraffin (or Tween 80), respectively. For the fresh nonwoven mats (control group, Figure 8A,D), subtle peaks were observed at 4.20 min, suggesting that these peaks were attributed to the adsorbate nicotine after the nonwoven mat had been used. The peak of nicotine could be separated and observed at 4.20 min with the UV absorption detection at 259 nm, as analyzed using the HPLC–UV system. The peak areas acquired from various samples were calculated (using the standard curves and linear equation) as 152.25 ± 6.59 µg (F–fGglp), 180.10 ± 16.08 µg (UV–F–fGglp), 101.15 ± 16.15 µg (F–fGgT), and 162.91 ± 17.23 µg (UV–F–fGgT; Table S3). Table S3 and Figure 8B,C,E,F clearly show that the content of adsorbed nicotine could be increased by 18–61% when the UV light was irradiated on the nonwoven mats during the sidestream smoke fumigation period. The peaks of the liquid paraffin and Tween 80 groups were notably present at 4.53 min. When Figure 8A,D was compared with the others, the peaks at 4.53 min in Figure 8B,C,E,F were stronger than Figure 8A,D as presented in Table S3. These results could be determined by the correlation of nicotine with liquid paraffin (or Tween 80). Noteworthily, because liquid paraffin and Tween 80 were not dissolvable by mobile phase methanol, only the nicotine could be dissolved, which suggested that part of the liquid paraffin and Tween 80 could be dissolved and extracted when interacting with nicotine. To further verify this hypothesis, the extraction liquid from the nonwoven mat was analyzed using both HPLC–UV and HPLC–MS data. The results of HPLC–MS provided the necessary information to precisely understand the varieties of nicotine derivatives that were captured or photodegraded from the nicotine in the sidestream cigarette smoke.

**Figure 9:**
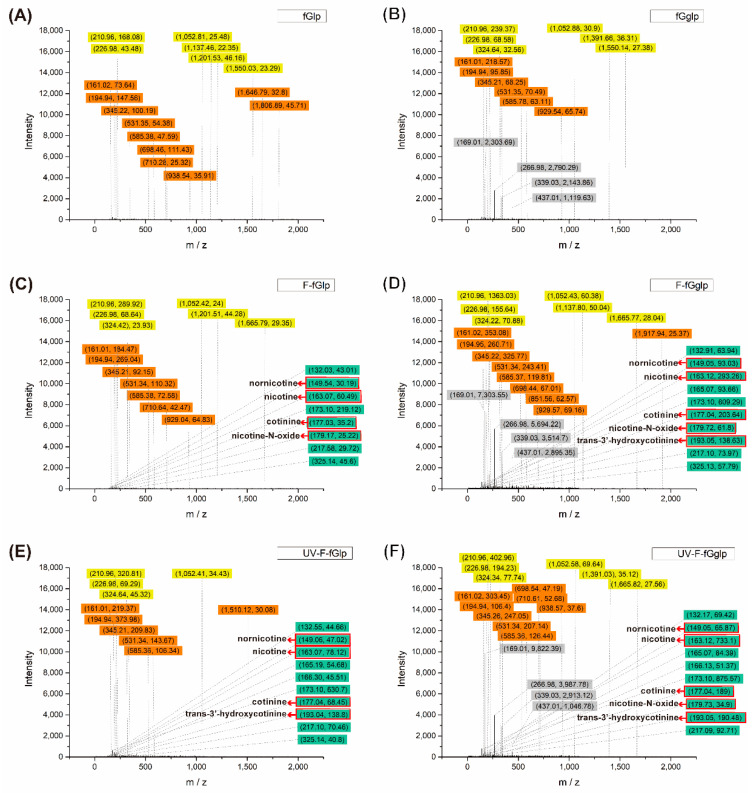
The HPLC–MS results of (**A**) fGlp, (**B**) fGglp, (**C**) F–fGlp, (**D**) F–fGglp, (**E**) UV–F–fGlp, (**F**) UV–F–fGglp (fG: fish gelatin, g: gallic acid, lp: liquid paraffin, F: fumigated, UV: 365 nm).

**Figure 10:**
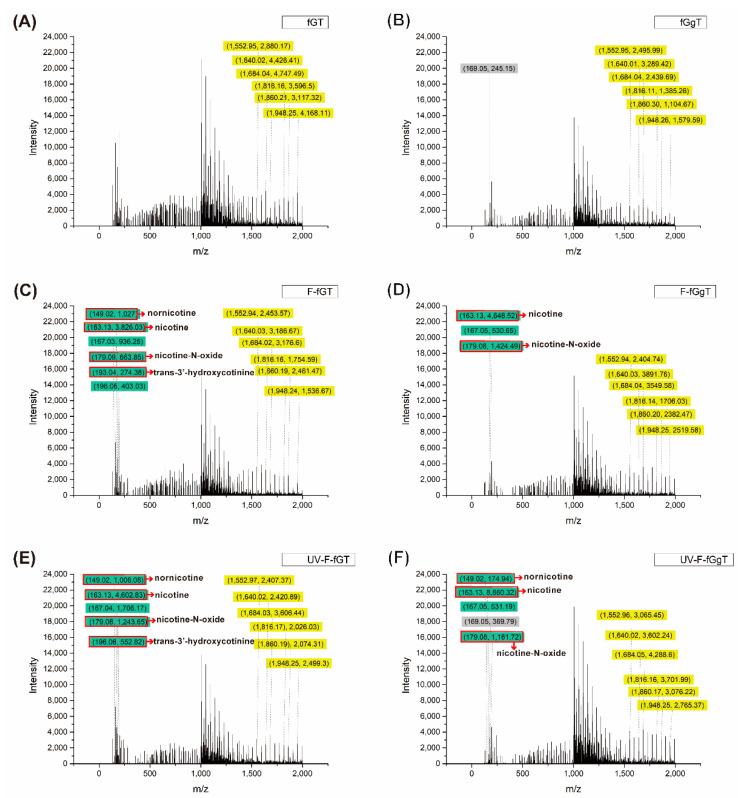
The HPLC–MS results of (**A**) fGT, (**B**) fGgT, (**C**) F–fGT, (**D**) F–fGgT, (**E**) UV–F–fGT, (**F**) UV–F–fGgT. (fG: fish gelatin, g: gallic acid, T: Tween 80, F: fumigated, UV: 365 nm).

The HPLC–MS result suggested that the intensity of nicotine was greater when the nonwoven mat was irradiated by UV light (Figure 9C–E and D–F, before and after). When the nonwoven mat was prepared with a liquid paraffin system (Figure 9), the HPLC–MS result indicated that both nicotine and nicotine derivative molecules could be extensively captured upon application of both gallic acid and 365 nm UV light. The nicotine derivatives observed were as follows: parent nicotine (*m*/*z* = 163), nornicotine (*m*/*z* = 149), nicotine-N-oxide (*m*/*z* = 179), cotinine (*m*/*z* = 177), and trans-3′-hydroxycotinine (*m*/*z* = 193), which were referred by references [3,4]. Notably, the inclusion of gallic acid in the nonwoven mat produced substantially greater adsorption of nicotine derivatives (Figure 9D,F). When the mat was irradiated with UV light, the amount of nicotine adsorbate was markedly higher and the intensity of nicotine derivatives (especially trans-3′-hydroxycotinine) was higher (Figure 9D,F). Figure 10 also displays the results for the fGgT nonwoven mat, which replaced liquid paraffin with Tween 80 (Figure 10D); when this nonwoven mat absorbed the smoke, we noted that the Tween 80 also dissolved the nicotine and the signal of the nicotine derivatives, (Figure 10E). However, when the nonwoven mat was irradiated by 365 nm UV light, the nonwoven mat could adsorb more smoke, which also enhanced the signal of nicotine derivatives, possibly due to the gallic acid-induced photodegradation (Figure 10F). In Figure 10F, the signal intensity of the norcotinine and nicotine-N-oxide derivatives are obviously increasing. The results also indicate a markedly higher nicotine intensity compared with that shown in Figure 10E. In general, these results may indicate that the gallic acid addition not only enhances the absorption property of the nonwoven mat but also enables it to exert a photodegradation function in conjunction with suitable UV light irradiation. In accordance with the fluorescence emission spectra and HPLC–MS results, we theorize that the fluorescence emission mechanism of the nonwoven mat was strongly tied to the chemical structure of the gallic acid and the nicotine molecules that were dissolved by liquid paraffin or Tween 80.

**Scheme 1:**
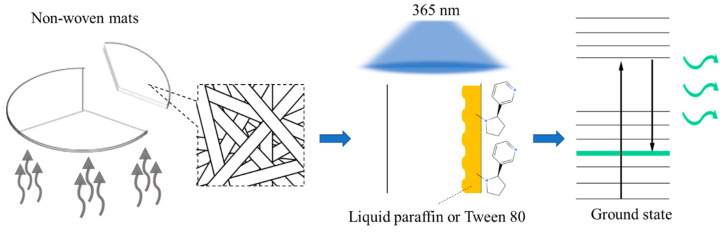
Mechanism of photoindication and photodegradation functions.

The mechanism of the photoindication function of the nonwoven mats could be explained by the fluorescence emission spectra. When the gelatin mat was irradiated by 365-nm UV light, a dominant purple emission peak was observed at approximately 400–439 nm, with the other shoulder peak observed at a blue emission wavelength of approximately 468 nm. This purple emission mixed again, concealed the 400–439-nm emission, and made the observed color present when close to navy blue light. Regarding another aspect of the photodegradation property, we discovered that, because nicotine is a greasy pollutant, the addition of nicotine-dissolving polymer materials (i.e., liquid paraffin or Tween 80) could help adsorb smoke and react with nicotine after the sidestream smoke fumigated the gelatin mat to raise the adsorption ability. Subsequently, the nicotine contained in the sidestream smoke caused the purple emission peak to exhibit redshift close to the 468-nm emission, and then changed the observed color from purple-blue to pale blue. According to the fundamental mechanism of the fluorescence property, the π-π* (orbital) of the conjugated system was a major transition type that contributed to the following fluorescence emission. After the nonwoven mat absorbed the nicotine, the conjugated system of gallic acid inside provided a transition path that produced the fluorescence. When the 365-nm UV light provided energy to the conjugated system to excite the electron, the electrons would then relax by releasing energy in the form of fluorescence and returning to the ground state with a different vibrational energy level. When the nonwoven mat was prepared using Tween 80 or liquid paraffin, more nicotine could be captured, and the fluorescence intensity was greater due to the increase in the conjugated system. Although gallic acid and UV light with an accurate absorption wavelength were the key parts of the nonwoven mat developed with photoindication and photodegradation, their addition in the electrospinning with gelatin also provide benefits for fiber and porous structure formation. The HPLC–UV results suggest that liquid paraffin and Tween 80 could be dissolved when they captured nicotine molecules. Conversely, no relevant elution peaks were observed in the chromatogram when the nonwoven mats were not fumigated with sidestream smoke. On the basis of the fluorescence spectra, we can speculate that the liquid paraffin and Tween 80 in this study captured nicotine that was adsorbing on the surface of fibers, exhibiting a noteworthy fluorescence phenomenon that was based on the excitation of the electrons of the conjugated system by 365-nm UV light. Moreover, gallic acid was excited, and part of the excited state was transformed from a singlet state to a triplet state. The triplet state of the gallic acid performed two functions: enhancement of the UV absorption and photodegradation of the pollutant [2]. The aforementioned mechanism is depicted in Scheme 1. Thereafter, when the nonwoven mats were soaked in methanol, a part of liquid paraffin and Tween 80 that combined with the nicotine was extracted and subsequently detected by HPLC–UV. The absorbed dose of nicotine was quantitively determined and is displayed in Table S3. Notably, the gallic acid could be leached out and detected. However, the amount of adsorbed nicotine was much greater than that of the added gallic acid. This result reveals that the remaining part of the nicotine was extracted by the nicotine-dissolving polymer in the fiber (i.e., paraffin or Tween 80). We also confirmed that the combination of 365-nm UV light, liquid paraffin or Tween 80, and gallic acid produced the two optimal parameter configurations. UV–F–fGglp and UV–F–fGgT nonwoven mats absorbed twice the dose of nicotine compared to the other groups. Obviously, gallic acid and nicotine-dissolving polymer work synergistically to capture more nicotine molecules. On the basis of the HPLC–MS results, we conclude that the gallic acid and UV light were associated with greater absorption of nicotine and simultaneously generated a series of nicotine derivatives when liquid paraffin and Tween 80 dissolved the nicotine and transferred it to the extract solution. We assert that the triplet state of gallic acid caused the photodegradation of nicotine to generate a series of nicotine derivatives. According to this evidence, we used a nonwoven mat that included gallic acid and a nicotine-dissolving polymer to adsorb smoke under 365-nm UV light irradiation. The HPLC–MS results showed the signals of cotinine (*m*/*z* = 177) and trans-3′-hydroxycotinine (*m*/*z* = 193), which is reportedly the major metabolite of nicotine [4]. Apart from adsorption and degradation, the gelatin-based nonwoven mats fabricated in this study provided filtration functions to capture the nicotine from sidestream smoke and activate the photoindication property through 365 nm UV light irradiation. As the nonwoven mat developed in this study was a freestanding film, a portable fan-type device was designed to actively channel the sidestream smoke to be filtrated and alert the user to the health risk of the surrounding air quality through the photoindication function and the 365 nm UV light. In Table S4, some of the pollutants adhered to the control plate with dust when the air was blown by the device, and then the control plate was cultured in an incubator for 48 hr. Almost 1064 units of bacterial colonies grew in the control plate. Conversely, there were not any bacterial colonies that appeared in the other agar plate. The result could prove the filtrate functionality of the nonwoven mats.

## 4. Conclusions

In this study, nonwoven mats composed of gelatin, gallic acid, and nicotine-dissolving polymers (liquid paraffin or Tween 80) were designed and developed to efficiently filtrate and adsorb nicotine, and nicotine-dissolving polymers were able to dissolve the nicotine from sidestream smoke, causing it to react with the gelatin structure and alerting users to the health risk of the air environment. Regarding the nicotine-dissolving polymers (liquid paraffin or Tween 80), they could not only capture the nicotine molecules, but also oxidize, dehydrogenate, and demethylate the nicotine to generate nicotine derivatives that included cotinine and trans-3′-hydroxycotinine. When those nonwoven mats were fumigated with sidestream smoke and simultaneously irradiated by 365-nm UV light, the adsorbed dose of nicotine increased by 18–61% compared with the mats not containing the gallic acid or nicotine-dissolving polymers.

## Data Availability

The data presented in this study are available on request from the corresponding author.

## Supplementary Materials

The following are available online at https://www.mdpi.com/article/10.3390/polym13234245/s1, Scheme S1: Experimental steps of the nonwoven mats fumigated with sidestream smoke, Scheme S2: Powerful yet compact blower attached to portable equipment, Scheme S3: Experimental steps of the pollute filtrate. (A) The combination of the wearable equipment and nonwoven mat, (B) The control plate, (C) The filter plate, Table S1: The porosity of nonwoven mat (fG: fish Gelatin, g: gallic acid, lp: liquid paraffin), Table S2: The porosity of nonwoven mat (fG: fish Gelatin, g: gallic acid, T: Tween 80), Table S3: The integral area and absorbed dose of experimental groups, Table S4: The intensity of nicotine derivatives in Figure 9C–F (fG: fish Gelatin, g: gallic acid, lp: liquid paraffin, F: fumigated, UV: 365 nm), Table S5: The intensity of nicotine derivatives in Figure 10C–F (fG: fish Gelatin, g: gallic acid, T: Tween 80, F: fumigated, UV: 365 nm).
