# Supplementary material for: Gallic Acid-Containing Gelatin-Based Nonwoven Mat with Synergistic Photodegradation and Photoindication Function for Reducing Nicotine
Source: Polymers (Basel). 2021 Dec 3;13(23):4245. doi: 10.3390/polym13234245 (PMC8659907; doi:10.3390/polym13234245)
Supplement: Supplementary file 1 [file polymers-13-04245-s001.zip › supporting information-SI.pdf]

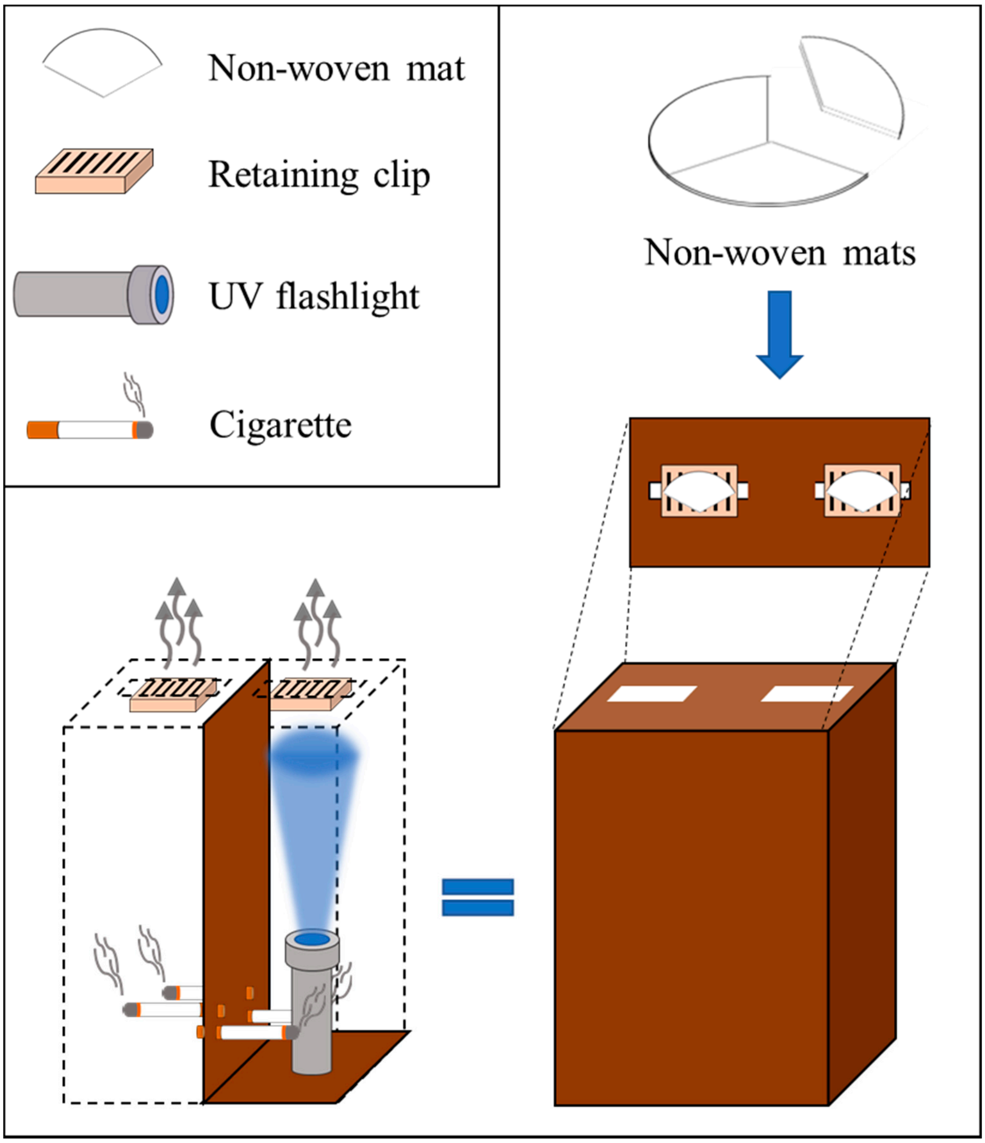

Schematic Diagram S1. Experimental steps of the nonwoven mats fumigated with sidestream smoke.

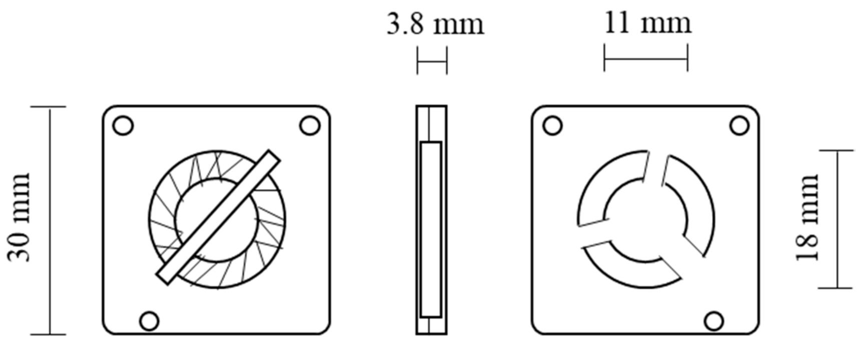

Schematic Diagram S2. Powerful yet compact blower attached to portable equipment.

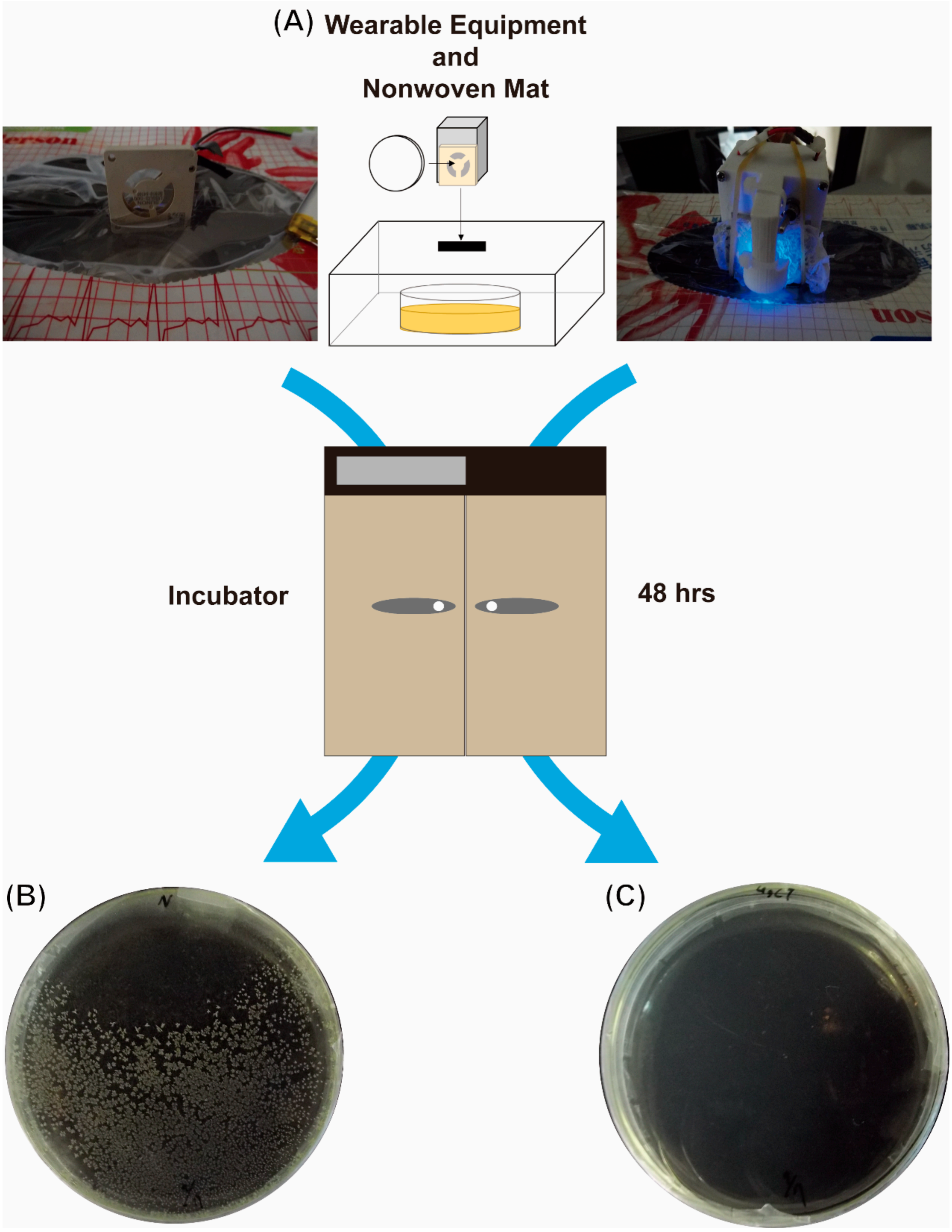

Schematic Diagram S3. Experimental steps of the pollute filtrate. (A) The combination of the wearable equipment and nonwoven mat, (B) The control plate, (C) The filter plate

Table.S1 The porosity of nonwoven mat (fG : fish Gelatin, g : gallic acid, lp : liquid paraffin)

Sample	Count	Total Area (μm^2)	Average (μm^2)	Standard Deviation	Porosity (%)
fG	972	259.69	0.27	0.40	14.84
fGg	1244	548.39	0.44	0.61	31.59
fGlp	965	193.48	0.20	0.38	7.51
fGglp	908	202.843	0.223	0.33547	11.566

Table.S2 The porosity of nonwoven mat (fG : fish Gelatin, g : gallic acid, T : Tween

Sample	Count	Total Area (μm^2)	Average (μm^2)	Standard Deviation	Porosity (%)
fG	622	36.31	0.06	0.08	18.32
fGg	976	40.68	0.04	0.04	20.58
fGT	308	10.83	0.03	0.03	5.49
fGgT	379	19.89	0.05	0.06	10.08

80)

Table.S3 The integral area and absorbed dose of experimental groups.

		fGglp	F-fGglp	UV-F-fGglp	fGgT	F-fGgT	UV-F-fGgT
**Gallic acid**	Leaching dose (**mg**)	1.97 ± 1.02	1.87 ± 0.27	1.90 ± 0.10	1.90 ± 0.94	2.02 ± 0.64	2.19 ± 1.09
**Nicotine**	Adsorbed dose (**μg**)	none	152.25 ± 6.59	180.10 ± 16.08	none	101.15 ± 16.15	162.91 ± 17.23
**Liquid paraffin**	Integral area (**mAU^2**)	257146 ± 17824.99	333085 ± 11895.65	345082 ± 10468.82			
**Tween 80**	Integral area (**mAU^2**)				107896 ± 4804.36	163457 ± 3729.37	190243 ± 8427.22

Table.S4 The intensity of nicotine derivatives in Figures 9C–F (fG : fish Gelatin, g : gallic acid, lp : liquid paraffin, F : fumigated, UV : 365 nm)

	**F-fGlp**	**F-fGglp**	**UV-F-fGlp**	**UV-F-fGglp**
**Nicotine (m/z = 163)**	60.49	293.26	78.12	733.10
**Nornicotine (m/z = 149)**	30.19	93.03	47.02	65.87
**Nicotine-N-Oxide (m/z = 179)**	25.22	61.80	none	34.90
**Cotinine (m/z = 177)**	35.20	203.64	68.45	189
**Trans-3'-Hydroxycotinine (m/z = 193)**	none	138.63	138.80	190.48

Table.S5 The intensity of nicotine derivatives in Figures 10C–F (fG : fish Gelatin, g : gallic acid, T : Tween 80, F : fumigated, UV : 365 nm)

	**F-fGT**	**F-fGgT**	**UV-F-fGT**	**UV-F-fGgT**
**Nicotine (m/z = 163)**	3826.03	4648.52	4602.83	8860.32
**Nornicotine (m/z = 149)**	1027	none	1006.08	174.94
**Nicotine-N-Oxide (m/z = 179)**	663.85	1424.49	1243.65	1161.72
**Cotinine (m/z = 177)**	none	none	none	none
**Trans-3'-Hydroxycotinine (m/z = 193)**	274.38	none	552.82	none
